# Response of Phytoplankton Photophysiology to Varying Environmental Conditions in the Sub-Antarctic and Polar Frontal Zone

**DOI:** 10.1371/journal.pone.0072165

**Published:** 2013-08-19

**Authors:** Wee Cheah, Andrew McMinn, F. Brian Griffiths, Karen J. Westwood, Simon W. Wright, Lesley A. Clementson

**Affiliations:** 1 Institute for Marine and Antarctic Studies, University of Tasmania, Hobart Tasmania, Australia; 2 Antarctic Climate and Ecosystems CRC, University of Tasmania, Hobart, Tasmania, Australia; 3 CSIRO Division of Marine and Atmospheric Research, Hobart, Tasmania, Australia; 4 Australian Antarctic Division, Channel Highway, Kingston, Tasmania, Australia; University of Connecticut, United States of America

## Abstract

Climate-driven changes are expected to alter the hydrography of the Sub-Antarctic Zone (SAZ) and Polar Frontal Zone (PFZ) south of Australia, in which distinct regional environments are believed to be responsible for the differences in phytoplankton biomass in these regions. Here, we report how the dynamic influences of light, iron and temperature, which are responsible for the photophysiological differences between phytoplankton in the SAZ and PFZ, contribute to the biomass differences in these regions. High effective photochemical efficiency of photosystem II (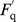/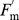 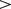
0.4), maximum photosynthesis rate (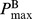), light-saturation intensity (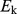), maximum rate of photosynthetic electron transport (1/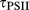), and low photoprotective pigment concentrations observed in the SAZ correspond to high chlorophyll 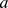 and iron concentrations. In contrast, phytoplankton in the PFZ exhibits low 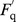/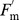 (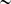 0.2) and high concentrations of photoprotective pigments under low light environment. Strong negative relationships between iron, temperature, and photoprotective pigments demonstrate that cells were producing more photoprotective pigments under low temperature and iron conditions, and are responsible for the low biomass and low productivity measured in the PFZ. As warming and enhanced iron input is expected in this region, this could probably increase phytoplankton photosynthesis in this region. However, complex interactions between the biogeochemical processes (e.g. stratification caused by warming could prevent mixing of nutrients), which control phytoplankton biomass and productivity, remain uncertain.

## Introduction

The Southern Ocean is the largest of the high nutrient, low chlorophyll (HNLC) regions due primarily to widespread iron limitation of phytoplankton [1], [2]. However, seasonal algal blooms are still observed in places such as South Georgia, Kerguelen Island, Crozet Islands, and the waters south of Australia [3], [4], [5], [6]. These blooms are generally confined to waters replete in iron. A strong lateral gradient in nutrient concentrations and phytoplankton biomass is often observed between the bloom area and surrounding waters. The ocean south of Tasmania is such an area where there is a consistently higher chlorophyll 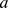 (chl 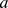) concentration in the east (SAZ-east, 150–160

E) of the Sub-Antarctic Zone (SAZ) than in the west (SAZ-west, 135–145

**Figure 1:**
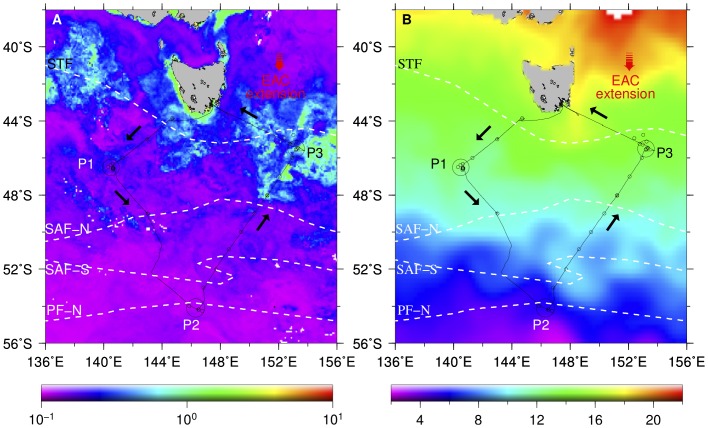
SAZ-Sense voyage track and sampling locations. Left panel (A) shows MODIS-Aqua 9-km resolution chlorophyll 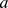 concentration (mg m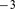) and right panel (A) show Reynolds sea surface temperature (°C) daily composite images from 17 January to 20 February 2007. Voyage track direction is shown by black arrows and black circles are the process stations (P1, process station 1; P2, process station 2; P3, process station 3). Subtropical front (STF), Sub-Antarctic front north (SAF-N), Sub-Antarctic front south (SAF-S), and polar front (PF) are the approximate locations of major oceanic fronts observed during the cruise. Red arrow indicates the approximate direction of the East Australian Current (EAC).

E) of the SAZ or in the Polar Frontal Zone (PFZ) [6] (Figure 1). Distinct regional physical processes are believed to be responsible for the variability in biogeochemical properties in these regions [7]. The SAZ-west region is typical of much of the circumpolar SAZ with relatively cold and less saline waters. In contrast, SAZ-east waters are warmer and saltier due to the influence of subtropical waters of the East Australian Current (EAC) [8]. Changes in climate are expected to alter the atmospheric and ocean circulation in this region, including the expansion of the subtropical gyres, and the southward extent of the EAC [9], [10]. However, the impact of these changes on biogeochemical properties and phytoplankton in this region is still uncertain.

Phytoplankton experiencing iron-deficiency have been shown to posses lower concentration of iron rich cellular components such as cytochrome 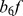 (cyt 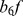) and photosystem I (PSI) protein complexes [11], [12] and higher concentration of photoprotective pigments [13], [14] than iron-replete cells. These adaptation mechanisms impact the photochemical efficiency of photosynthesis and result in reduced growth rates and photochemical efficiency of photosystem II (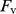/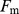) [15]. In addition to iron, light also plays a critical role in phytoplankton production in the Southern Ocean [16]. The presence of strong winds causes the formation of deep mixed layers and vertical mixing [2]. This leads to rapid fluctuation in irradiance with supraoptimal irradiance occuring near the surface and complete darkness below euphotic zone, factors which impose a great challenge on the photophysiological dynamics of phytoplankton. Contrasting photoacclimation strategies, such as maximizing light harvesting capacity under low irradiance, while employing photoprotective mechanisms under excessive irradiance, are required to respond to these rapid irradiance fluctuations [17], [18].

It has been suggested that under iron-light co-limitation conditions, photosynthesis is ultimately limited by light, but the production of light harvesting protein-complexes (e.g. photosystem II (PSII) and PSI) is constrained by iron availability [19]. In contrast, under low iron, high light conditions, photoinhibition or photodamage may occur as iron limitation decreases the synthesis of cyt 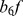 complexes, which are required in the activation of photoprotective mechanisms [12], [18]. Thus, iron-limited cells are less efficient at coping with an environment with rapid irradiance fluctuations than iron-replete cells [12], [18], [20]. Furthermore, other abiotic factors such as temperature, may also play an important role in defining the photophysiological state of phytoplankton. When combined with other factors such as nutrients or light, temperature can has a synergistic or antagonistic effect on phytoplankton physiology [14], [21].

In this study we show the photophysiological response of natural phytoplankton communities at three contrasting sites with distinct hydrological, chemical, and biological properties in the SAZ and PFZ based on data obtained during the SAZ-Sense (Sub-Antarctic Zone Sensitivity to environmental change) cruise aboard *RSV Aurora Australis* from 17 January to 20 February 2007. The aim of the SAZ-Sense expedition (43-55 S, 140-154 E) was to investigate the interactions between biogeochemical forcing and microbial ecology at three contrasting sites in the highly dynamic SAZ and PFZ systems under the predicted influence of major currents (e.g. EAC and Antarctic Circumpolar Current) [22]. In this study, we analyze the effects of iron, light and temperature on phytoplankton photophysiology based on high definition vertical and horizontal field observations and identify the parameters that are causing the difference in biomass and production rates in the SAZ and PFZ.

## Materials and Methods

### Ethics Statement

No specific permits or permissions were required as field studies were carried out in international waters and did not involve endangered or protected species.

### Hydrography

The SAZ-Sense cruise covered an area south of Tasmania with contrasting water masses ranging from subtropical to polar regions, and crossed several fronts (Figure 1). The five-week cruise consisted of 24 stations, three of which were selected as process stations (P1, P2, and P3, respectively) based on their hydrographic properties [23]. P1 in the SAZ-west is subjected to mixing between the polar front waters to the south and subtropical waters to the north, which influenced by the Indian Ocean sourced Leeuwin Current, and has low phytoplankton biomass representing much of present day SAZ. P2 is a typical PFZ consisted of polar waters from the Antarctic Circumpolar Current, whereas P3 in the SAZ-east is a SAZ currently experiencing the influence of subtropical waters from the EAC and has higher phytoplankton biomass than P1 and P2 [22]. Five to seven days were spent at each process station to allow repetitive sampling at these stations. Vertical profiles of hydrographic data were collected using a Seabird 911plus conductivity, temperature, and depth (CTD) sensor. Water samples for nitrate+nitrite, phosphate, silicate, photosynthesis-irradiance incubations, total particulate absorption, and phytoplankton pigments analyses were collected at discrete depths using 10 L Niskin bottles attached to the CTD rosette. A separate trace metal clean rosette was deployed alongside the main CTD rosette for sampling of trace metals [24]. Sampling for trace metals were carried out mostly in the process stations and some stations along transect. At each sampling, seawaters were collected at discrete depth from 15 m to 1000 m. The frequency and sampling depth intervals varied between stations. Dissolved iron (dFe) was determined on board using flow injection analysis with chemiluminescence detection. See [25], [24] for detailed sampling and analyses of iron.

Incident photosynthetically active radiation (PAR) was recorded continuously by the shipboard weather observatory using a LI-COR sensor and averaged over 10 minute intervals. The mixed layer depth (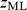) was calculated from each CTD profile as the shallowest depth at which the density was 0.05 kg m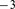 greater than at a depth of 8 m and using the Brunt-Väisälä frequency analysis [23]. The depth of the euphotic zone (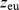) was calculated as 1% of the surface PAR [26]. Mean light levels in the mixed layer (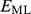) at three process stations were calculated as: 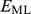 = 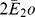
[1-e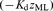]/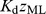 [27], [28]. 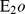 is the daily surface PAR. Vertical light attenuation coefficient (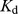) was calculated through linear regression of PAR data with depth as detailed in [26].

### Fast Repetition Rate Fluorometry

A Fasttracka Fast Repetition Rate (FRR) fluorometer (Chelsea Technology Group, UK) equipped with dual measuring chambers (dark and light) was deployed every second to third station and more frequent at each process station (Table S1) in profiling mode immediately after a CTD cast at a vertical rate of 0.5 m s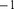 to acquire single turnover fluorescence transients. The light chamber measured fluorescence yields under natural ambient light while the dark chamber provided a short period (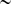 1 s) of dark-adaptation. FRR fluorescence yields were measured using a flash sequence consisting of a series of 100 subsaturation flashlets (1.1 *µ*s flash duration and 2.8 *µ*s inter flash period) and a series of 20 relaxation flashlets (1.1 *µ*s flash duration and 51.6 *µ*s inter flash period). Fluorescence transients obtained at each deployment were then fitted to the biophysical model of [29] to determine the maximum (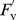/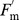), effective (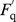/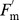) photochemical efficiency of PSII under ambient light and maximum photochemical efficiency of PSII in darkness (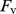/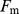), and functional absorption cross section of PSII in darkness (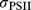) and under ambient light (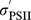), as described by [30]. The photochemical quenching coefficient, 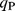, was estimated from 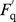/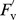 = (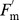-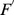)/(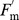-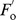) [31], [32]. Table 1 shows the descriptions of the parameters used in this study.

**Table 1:** Photosynthetic parameters and definitions used in this study.

Parameter	Definition	Units
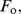 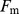	Mininum and maximum fluorescence in dark-adapted state	Dimensionless
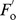 , 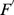 , 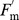	Minimum, steady state and maximum fluorescence under ambient light	Dimensionless
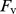 / 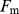	Maximum photochemical efficiency, ( 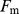 - 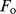 )/ 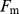	Dimensionless
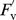 / 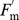	Maximum photochemical efficiency under ambient light, ( 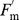 - 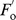 )/ 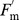	Dimensionless
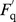 / 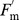	Photochemical efficiency under ambient light, ( 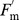 - 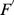 )/ 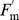	Dimensionless
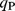	Photochemical quenching or PSII efficiency factor under ambient light	Dimensionless
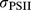 , 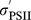	Functional absorption cross section of PSII in dark-adapted state and under ambient light	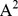 quanta 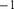
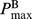	Light saturated photosynthesis rate	mg C (mg chl 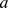 ) 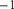 h 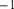
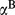	Light limited photosynthesis rate	mg C (mg chl 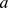 ) 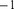 h 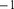 (*µ*mol photons m 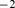 s 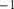 ) 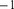
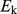	Light saturation index	*µ*mol photons m 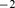 s 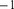
1/ 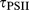	The maximum rate of photosynthetic electron transport from charge separation to carbon fixation	ms 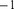
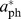	Chl 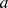 -specific phytoplankton absorption coefficient of phytoplankton	m 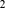 (mg chl 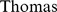 ) 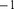

Surface fluorescence signals (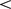 10 m) were corrected for background fluorescence using filtered seawater collected at 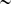 6 m depth. Fluorescence correction for greater depths was not carried out as a sample blank was not available. The FRR fluorometer was integrated with both PAR and pressure sensors. The vessel was aligned to avoid ship shadow prior to each FRR fluorometer deployment.

### Photosynthesis-Irradiance Incubations

Photosynthesis-irradiance (P-E) incubations were carried out every second to third station and at least twice at each process station. Incubations were based on small bottle 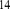
C incubation technique [33] as detailed in [26]. In brief, at each selected sampling station, 400 mL of seawaters were collected from six to seven depths ranging from surface to 125 m and stored in darkened polycarbonate jars in a seawater-cooled, insulated container, until the commencement of incubation. Before incubation, each jar was spiked with 6.327×10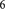 Bq (0.171 mCi) NaH
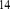
CO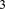 to produce a working solution of 39.183×10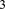 Bq mL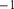 (1.1 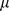
Ci mL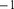). Seven mL aliquots of working solution were then added to transparent glass scintillation vials and incubated for 1 hour at 21 light intensities. After acidification and venting to remove excess inorganic carbon, 10 mL Aquassure scintillation fluids were added to each vial and shaken. Samples were then counted using a Packard TriCarb 2900TR scintillation counter with the maximum counting time set at 5 min.

Maximum photosynthetic rate, 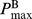 [mg C (mg chl 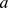)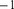 h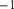], initial slope of the P-E curve, 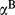 [mg C (mg chl 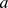)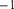 h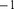 (*µ*mol photons m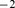 s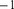)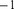] and the photoinhibition parameter, 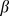 [mg C (mg chl 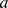)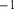 h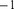 (*µ*mol photons m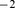 s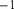)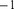] were then derived from P-E data based on the method of [34]. The maximum rate of photosynthetic electron transport from charge separation to carbon fixation (1/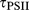) was calculated from the product of the P-E estimates of saturation irradiance, 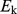 (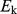 = 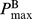/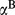) and the FRR fluorometry estimates of 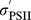 (1/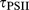 = 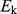
×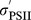) [35]. 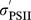 and 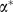 values were not scaled to the *in situ* light field.

### Particulate Absorption

Sampling and analysis of particulate absorption were carried out as per [36]. Briefly, 1–2 L of seawaters from two to six depths ranging from surface to 100 m were removed from appropriate Niskin bottles. Collected samples were then filtered under low vacuum (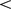 0.5 atm.) onto 25 mm Whatman GF/F filters and then immediately stored in liquid nitrogen until analysis. Total particulate [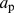 (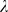)] and detrital [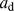 (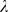)] matter absorption spectra were measured between wavelengths of 350 and 800 nm using a GBC 916 UV/VIS dual beam spectrophotometer equipped with an integrating sphere. Chl *a*-specific phytoplankton absorption spectra [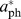
(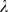), m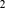 (mg chl 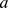)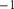] were obtained as the difference between the 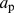 and 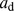 and normalized to chl 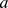 concentrations [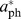 = (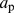 - 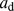)/chl 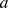].

### Phytoplankton Pigments and Community Structure

Seawater samples (1–2 L) were collected from three to eleven depths ranging from surface to 200 m and filtered under low (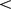 0.5 atm) on 13 mm Whatman GF/F filters. Filtered samples were then immediately stored in liquid nitrogen until analysis. Pigments were extracted by beadbeating the filters in 300 *µ*L dimethylformamide plus 50 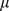
L methanol solution (containing 140 ng apo-8'-carotenal (Fluka) internal standard) and analyzed using a HPLC system (Waters 626 pump, Gilson 233xL autoinjector, Waters Symmetry C8 column, Waters 996 diode array detector, Hitachi FT1000 fluorescence detector) based on the method of [37]. Identification of pigments was carried out by comparing their retention times and absorption spectra with a sample of mixed standards from known cultures. Estimates of phytoplankton classes were determined using the CHEMTAX program and the pigment ratios from [38] and [39] as starting points. Data were split into five bins according to sample depth to allow for variation of pigment ratios according to irradiance. The depth bins and sample numbers in each bin were 0–21 m (75), 21–48 m (91), 48–85 m (104), 85–130 m (85), 130–250 m (119) (total = 474). In each of the six CHEMTAX runs, 40 sets of randomized pigment tables were applied to each depth bin to avoid the possibility of poor starting choices producing unrepresentative results. These tables were produced by randomly adjusting each of the initial pigment ratios by up to 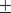
35% of the original ratio, as described in [39]. Results producing the lowest residual errors were compiled for subsequent interpretation.

### Statistics

Non-parametric Kruskal-Wallis one-way ANOVA was used to test the differences in parameters between process stations. Relationships between hydrological parameters (e.g. iron, temperature, light) and photosynthetic parameters (e.g. 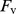/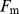, 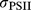, xanthophyll pigment concentrations) were determined using non-parametric Kendall's rank correlation (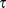) analysis based on collocated data (according to station and depth) from the entire data set.

## Results

### Hydrographic Conditions

**Figure 2:**
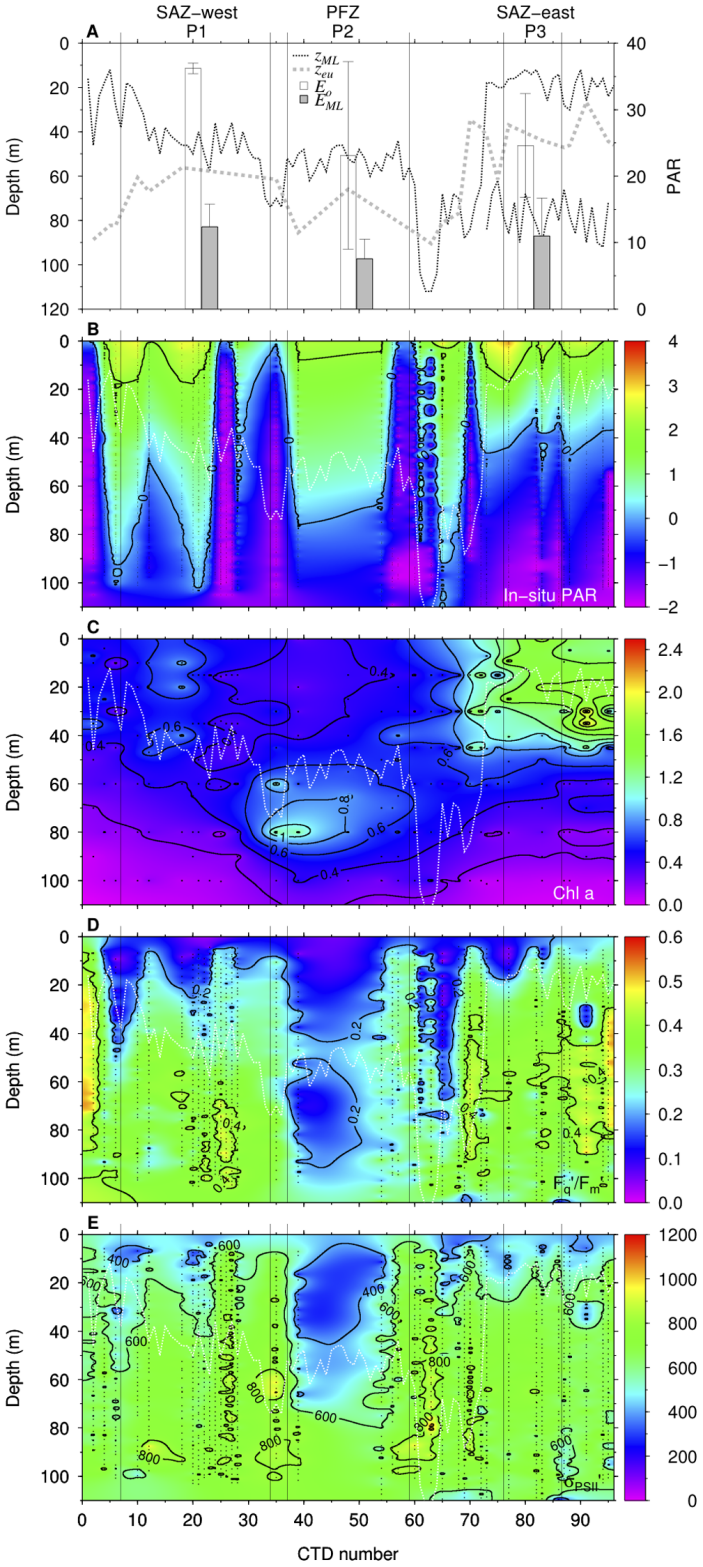
Profiles of mixed layer, euphotic depth, PAR, chl *a*, 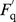/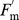, and 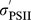. Panel A shows profiles of mixed layer depth (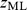, m), euphotic depth (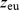, m) for the whole cruise, and mean surface PAR (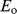, mol photons m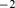 d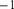) and mean PAR in the mixed layer (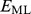, mol photons m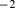 d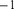) at P1, P2, and P3. Contour plots of (B) log (base-10) transformed *in situ* PAR (*µ*mol photons m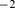 s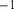), (C) chl 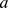 (mg m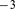), (D) effective photochemical efficiency of PSII (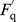/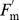, dimensionless), and (E) functional absorption cross section of PSII (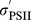, 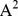 quanta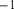). White lines indicate the mixed layer depth.

Sea surface temperature (SST, 10 m) in this study ranged from 5.0 
C observed at process station 2 (P2) in the PFZ to 17.3 
C observed in the subtropical region near process station 3 (P3). Mean seawater temperature from surface to 100 m was lowest at P2 (3.79
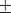
0.10 °C), and approximately three-fold higher at P1 (11.59
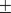
0.45 °C) and P3 (12.05
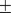
0.14 
C) than P2. Mean temperature at P2 differed significantly (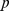 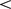 0.05) from P1 and P3. Sea surface salinity (10 m) in this study ranged from 33.79–35.53. The mixed layer depth (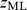) ranged from 12–112 m. 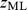 at process station 1 (P1) varied from 18–74 m, while at P2 a more constant 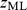 ranging from 44–62 m was recorded. At process station 3 (P3), a shallow mixed layer from 12–26 m overlaid a secondary pycnocline from 68–90 m. The depth of the euphotic zone (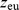) ranged from 34–90 m and 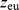 were mostly deeper than 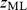 except for stations with a deep mixed layer (Figure 2A). Daily surface PAR (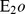) was higher at P1 (36.2
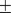
0.8 mol photons m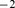 d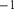) than P2 (23.1
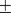
14.1 mol photons m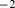 d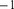) and P3 (24.6
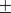
7.8 mol photons m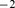 d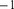) [26]. Mean PAR levels in the mixed layer (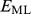) were higher at P1 (12.4
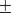
3.4 mol photons m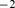 d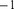) and P3 (11.0
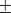
2.9 mol photons m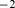 d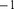) than P2 (7.6
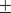
5.7 mol photons m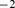 d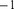) (Figure 2a). 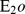 and 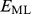 were not significantly different (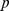 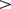 0.05) between process stations. High surface (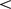 10 m) *in situ* PAR was observed at P3 (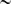
700 *µ*mol photons m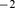 s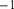) compared to P1 (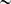
400 *µ*mol photons m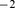 s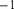) and P2 (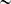
200 *µ*mol photons m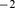 s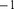) (Figure 2B).

**Figure 3:**
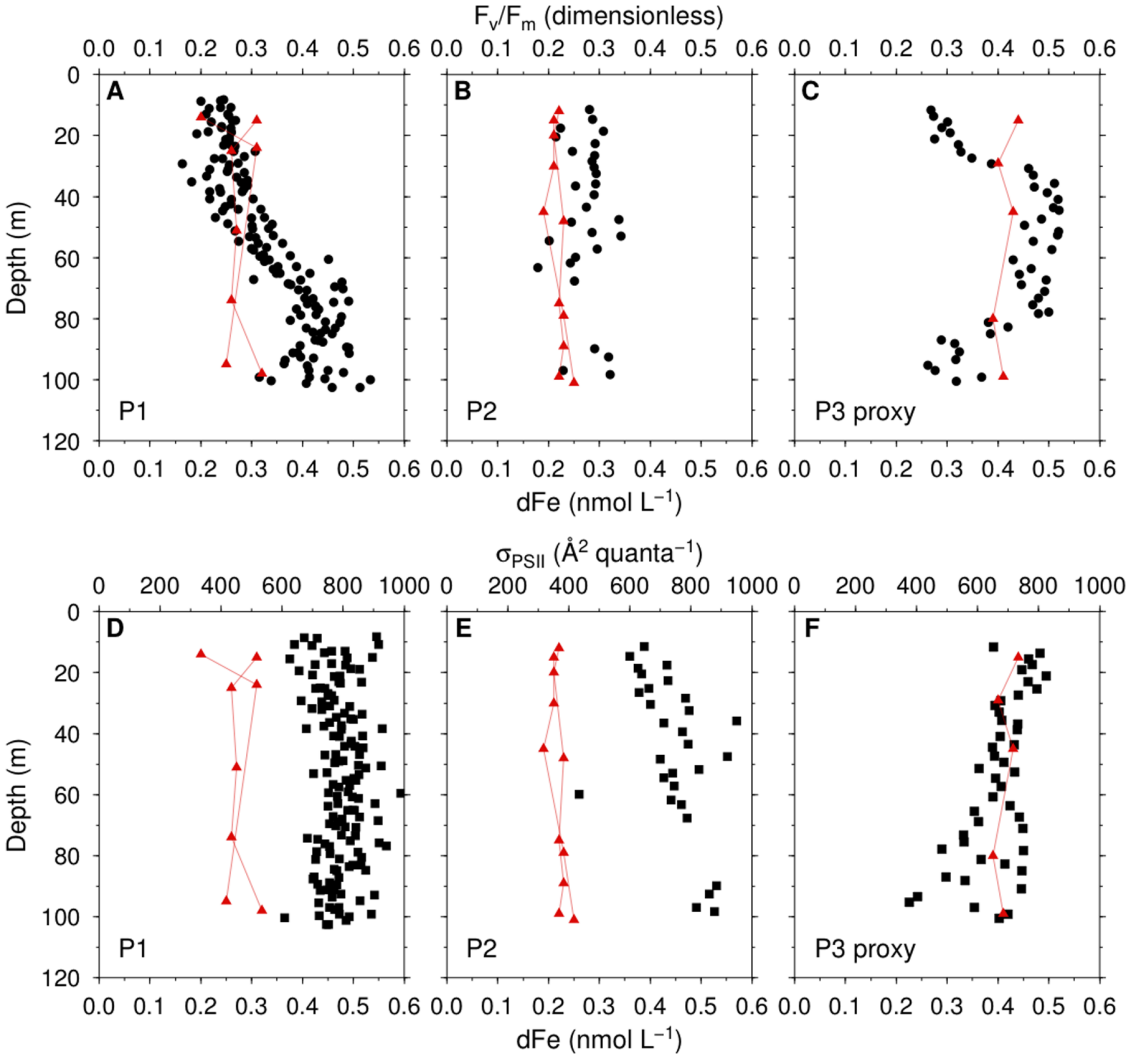
Night-time values of chlorophyll fluorescence parameters. Maximum photochemical efficiency (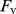/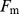, black circles) and dissolved iron (dFe, red triangles) at P1, P2 and P3 proxy (a station near P3) (A–C). Functional absorption cross section of PSII (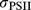, black squares) and dFe at P1, P2 and P3 proxy (D–F). The data at P1 and P2 were from two independent casts and data at P3 proxy were from one cast. dFe data were obtained from [24].

Surface chl 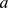 concentrations at P1 and P2 were generally 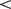 1.0 mg m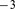, while at P3 values were 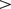 1.0 mg m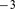. Chl 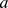 concentrations within the mixed layer were higher at P3 and P1 than P2. In contrast, a deep chl 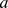 maximum was found at 60–80 m at P2 (Figure 2C) [40]. Concentrations of nitrate+nitrite and phosphate increased from north (subtropical zone, STZ) to south (PFZ) and with depth. Mixed layer nitrate+nitrite concentrations at P1 averaged at 5.2
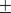
1.0 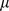
mol L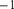, whilst P2 recorded a mean value of 24.3
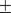
0.8 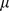
mol L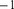 and 3.1
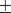
0.5 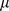
mol L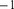 at P3. Mean phosphate concentrations in the mixed layer at P1, P2, and P3 were 0.41
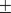
0.14 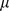
mol L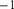, 1.57
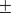
0.05 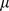
mol L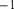, and 0.37
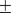
0.05 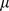
mol L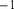, respectively. In contrast, silicate concentrations within the mixed layer were less than 1 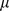
mol L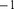 at all three stations (0.19
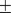
0.17 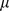
mol L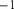 at P1, 0.74
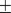
0.15 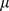
mol L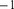 at P2, and 0.69
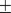
0.08 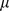
mol L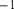 at P3) [23]. Maximum dissolved iron (dFe) concentrations were observed at P3 in SAZ-East and were lowest at P2 in the PFZ [25], [24]. Mean dFe concentrations within the mixed layer (0–40 m) at P1, P2 and P3 were 0.27
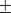
0.05, 0.21
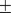
0.01, and 0.45
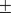
0.08 nmol L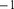, respectively. Below the mixed layer (40–100 m), dFe concentrations at P1 and P2 were similar to the mixed layer (0.28
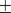
0.03 nmol L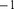 at P1 and 0.22
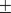
0.02 nmol L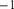 at P2), but decreased slightly at P3 (0.39
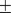
0.06 nmol L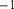) (Figure 3) [24]. Mean dFe concentrations within and below the mixed layer at each process station were significantly (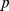 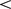 0.05) different.

### Photosynthetic Parameters

The effective photochemical efficiency of PSII under ambient irradiance (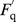/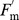) ranged from 0.02 to 0.54. 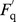/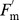 were low within the mixed layer but increased below the mixed layer except at P2, where low 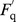/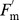 values extended beneath the mixed layer (Figure 2D). The vertical trend of the functional absorption cross section of PSII under ambient light (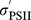) had a similar distribution to the 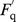/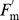 with low values within the mixed layer and increased below the mixed layer (Figure 2E). 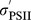 varied from 194 to 1128 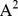 quanta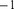. Both 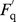/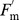 and 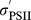 displayed a significant negative correlation with day-time *in situ* PAR (Table 2).

**Table 2:** Kendall's rank correlations between bio-optical properties and environmental variables.

Parameters	Correlation		
	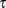	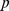	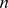
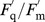 vs. *in situ* PAR 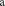	**−0.58**	0.00	80
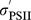 vs. *in situ* PAR 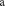	**−0.54**	0.00	85
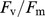 vs. temperature 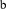	**0.40**	0.00	67
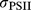 vs. temperature 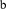	**−0.17**	0.01	108
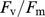 vs. dFe	**0.28**	0.02	37
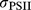 vs. dFe	0.03	0.79	37
dd+dt 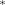 vs. *in situ* PAR 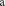	**0.54**	0.00	87
dt/(dd+dt) vs. *in situ* PAR 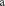	**0.50**	0.00	87
dd+dt^*^ vs. dFe	**−0.40**	0.01	22
dt/(dd+dt) vs. dFe	0.09	0.63	21
dd+dt^*^ vs. temperature 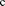	**−0.41**	0.00	40
dt/(dd+dt) vs. temperature 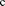	0.00	0.98	40

Correlations were perform on collocated data according to station and depth from the entire data set observed at all stations. Significant correlations at 95% significance level are indicate in **bold**.

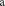
Only day-time values were used.

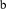
Only data below 40 m were used to reduce the influence of irradiance at surface.

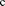
Only surface values (0–20 m) were used to avoid the influence of low irradiance at deeper depth.

**Figure 4:**
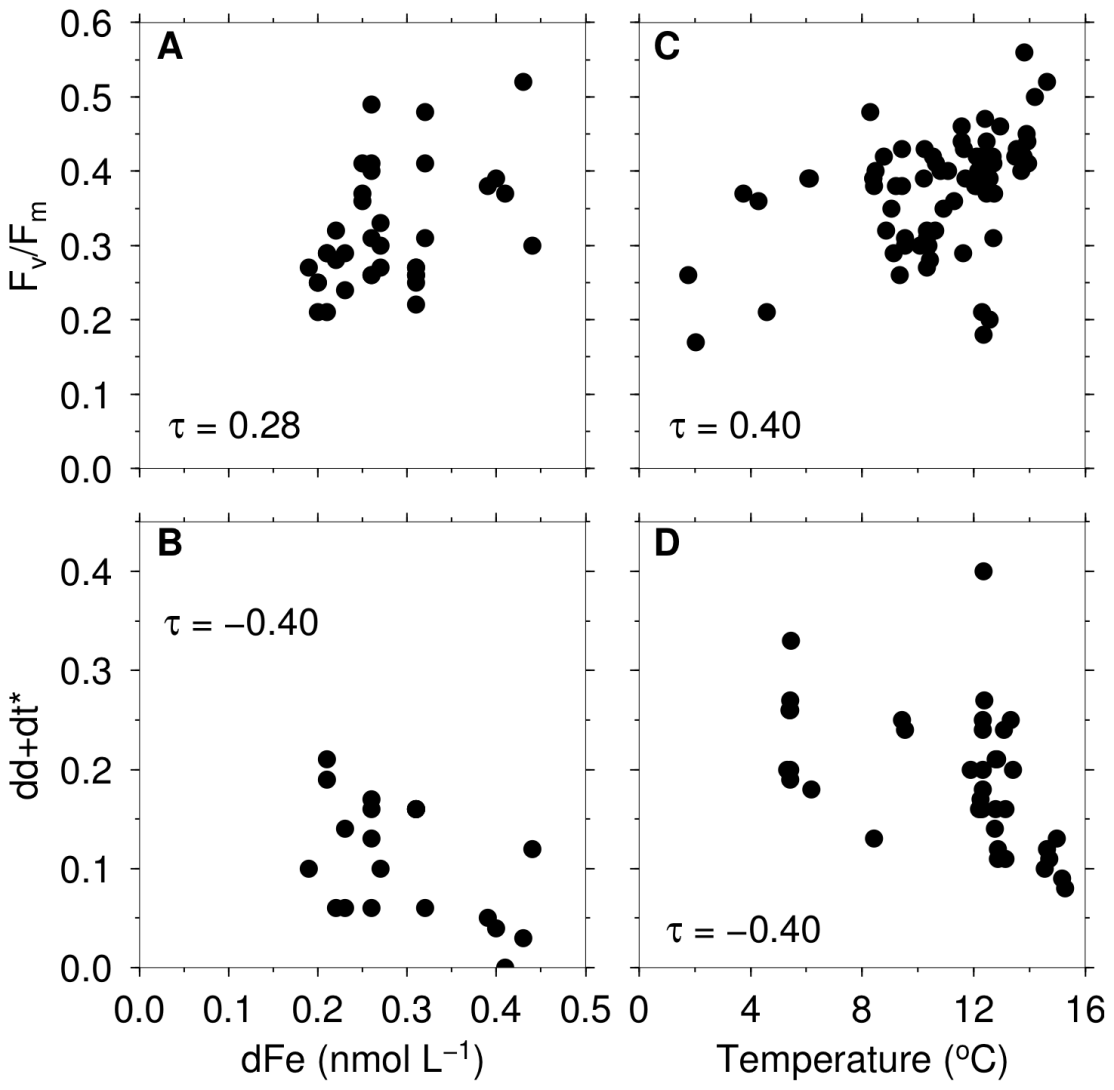
Kendall's rank correlations between dFe, 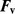/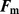, dd+dt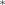, and temperature. Relationships between (A) dFe and 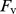/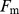, (B) dFe and dd+dt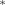, and between (C) temperature and 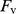/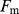 for data below 40 m. Relationship between (D) temperature and dd+dt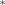 for surface (0–20 m) data. Kendall's rank correlation coefficients (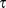) at 95% significance levels are shown.

Maximum photochemical efficiency of PSII measured at night (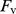/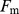) at P1 were low in the surface and increased with depth reaching its maximum (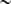 0.5) around 80 m (Figure 3A). At P2, 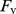/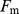 values were between 0.2–0.3 from the surface to 100 m (Figure 3B). At CTD 95 (P3 proxy), which was close to P3, 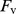/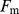 values were low in the mixed layer and increased to 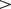 0.5 from 40–80 m dropping to 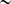 0.2 at 100 m (Figure 3C). Low surface 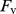/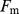 at this station could be due the shallow mixed layer, in which cells were more exposed to light during daytime. As the measurements of 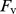/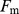 were carried out right after sunset, some PSII reaction centers (RCIIs) could still be in the reduced state. There was no night deployment of FRR fluorometer at P3. Night-time values of the functional absorption cross section of PSII (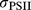) were higher at P1 and P2 than at P3 proxy. They were typically invariant with depth at P1, while increased at P2 and decreased at P3 (Figure 3D–F). 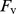/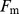 was significantly correlated with dFe and temperature (Figure 4), whereas a non-significant correlation was observed between dFe and 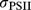. 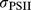 has a significant but weak negative correlation with temperature (Table 2).

**Figure 5:**
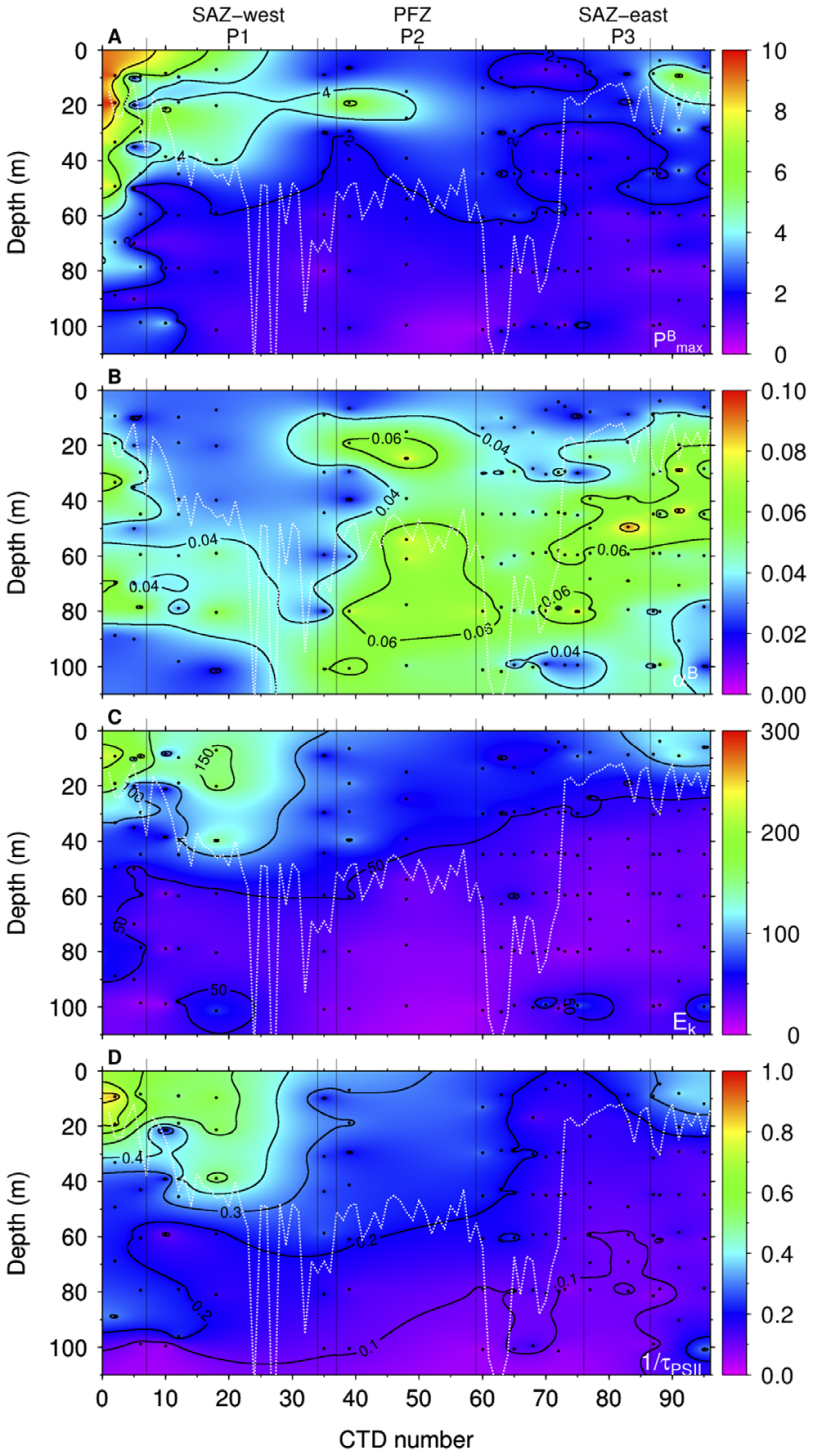
Profiles of photosynthetic parameters. Contour plots of (A) light saturated photosynthesis rate [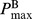, mg C (mg chl 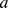)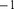 h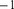], (B) initial slope of the P-E curve [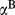, mg C (mg chl 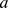)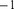 h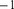 (*µ*mol photons m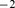 s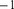)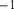], (C) saturation irradiance (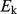, *µ*mol photons m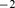 s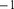), (D) maximum rate of photosynthetic electron transport from charge separation to carbon fixation (1/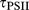, ms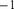).

P-E parameters were strongly influenced by mixed layer depth within the survey area with high 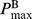 and 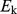 observed at the surface at P1 and stations closer to Tasmania (Figure 5A & C). Mean mixed layer 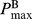 at P1, P2 and P3 were 4.8
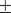
1.3, 3.2
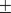
1.9 and 2.0
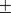
0.4 mg C (mg chl 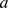)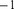 h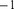, respectively. Mean mixed layer 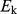 at P1 was 121.3
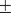
55.0 *µ*mol photons m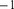 s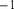, almost two-fold higher than at P2 (79.9
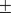
25.0 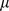
mol photons m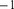 s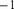) and P3 (79.1
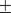
6.1 *µ*mol photons m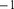 s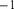) [26]. Vertical profiles of 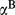 were different from 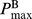 and 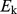, in which high values were generally observed at greater depths (Figure 5B). The maximum rate of photosynthetic electron transport (1/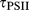) within the mixed layer was higher at P1 than P2 and P3, similar to 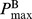 and 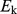 distributions (Figure 5D). 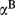, 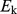, and 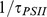 within and below the mixed layer were not significantly different (0.05) between stations. In contrast, within and below mixed layer were significantly different ( 0.05) between stations.

### Phytoplankton Pigment, Absorption and Community Structure

**Figure 6:**
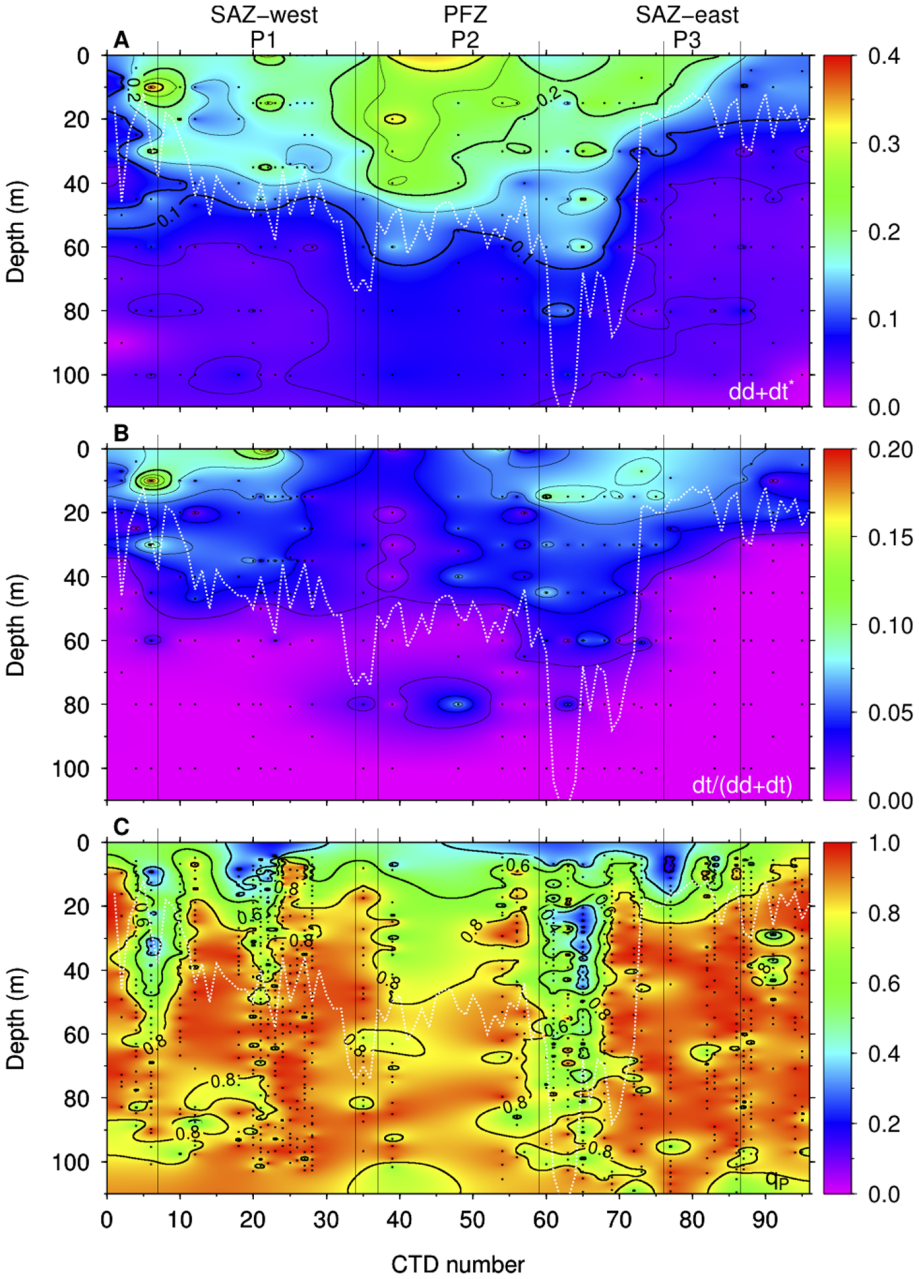
Profiles of photoprotective pigments and photochemical quenching. Contour plots of (A) the sum of diadinoxanthin and diatoxanthin normalized to chl 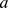 (dd+dt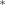), (B) the de-epoxidation state of the xanthophyll cycle [dt/(dd+dt)], and (C) photochemical quenching (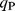). White lines indicate the mixed layer depth.

**Figure 7:**
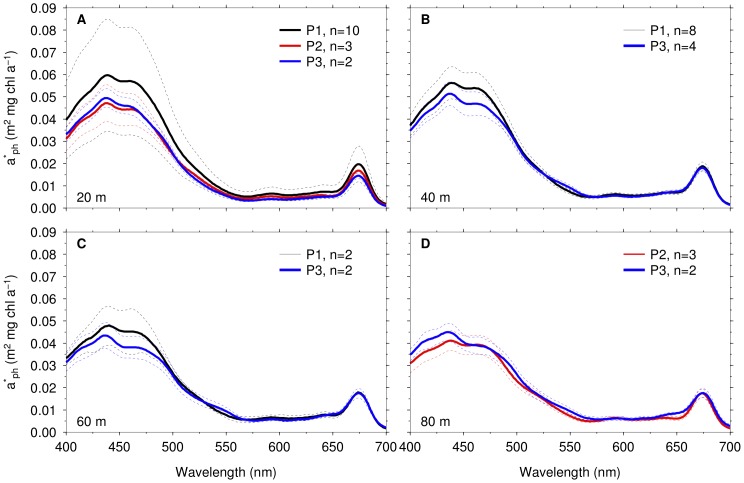
Depth profiles of mean chl 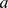-specific absorption coefficient of phytoplankton (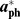) at process stations. 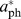
 at (A) 20 m, (B) 40 m, (C) 60 m, (D) 80 m. Dotted lines indicate the standard deviations.

Chlorophyll 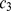 (chl 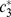) and fucoxanthin (fuco^*^) normalized to chl 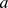 generally increased with depth at all sites (Table 3). No consistent pattern with depth in 19'-hexanoyloxyfucoxanthin (19'-hex^*^) and 19'-butanolyoxfucoxanthin (19'-but^*^) normalized to chl 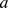 were observed between stations. Total photoprotective pigments of the xanthophyll cycle, represented by the sum of diadinoxanthin (dd) and diatoxanthin (dt) normalized to chl 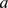 (dd+dt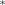), was higher at P2 than at P1 and P3. In contrast, the de-epoxidation state of the xanthophyll cycle, expressed as the amount of dt divided by the sum of dd and dt, dt/(dd+dt), was higher at P1 than at P2 or P3 (Fig. 5). Both dd+dt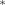 and dt/(dd+dt) showed a decrease with depth at all stations except for P2, where an increase in dt/(dd+dt) was observed below the mixed layer. At most stations, a strong gradient in xanthophyll pigments was observed within the mixed layer (Figure 6), whereas the patterns below the mixed layer were quite uniform except at P2. Both dd+dt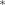 and dt/(dd+dt) displayed a significant correlation with light intensity (Table 2). A significant negative correlation was observed between dd+dt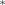 and dFe (Figure 4). In contrast, correlation between dt/(dd+dt) and dFe was not significant. Similar results were also found between xanthophyll pigments and temperature at the surface (0–20 m), in which a negative correlation was observed between dd+dt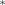 and temperature (Figure 4), whereas the relationship between dt(dd+dt) and temperature was not significant (Table 2). The chl 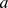-specific absorption coefficient of phytoplankton (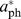) generally decreased with depth and was higher at P1, but not significantly different between P2 and P3. High values of standard deviations observed at the surface at P1 could be due to high sampling frequency at this station. High values of 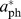 were observed at 437 nm and a secondary peak around 460 nm (Figure 7).

**Table 3:** Total chlorophyll 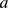 concentrations (mg m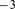) and accessory pigment composition normalized to chl 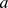 (denoted with asterisks) with depth at each station.

Station	Depth (m)	Chl 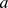	Chl 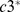	Fuco 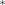	19'-Hex 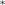	19'-But 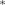	DD 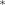	DT 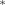
P1	20, 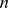 = 15	0.51 (0.22)	0.068 (0.009)	0.077 (0.033)	0.636 (0.081)	0.074 (0.007)	0.174 (0.044)	0.010 (0.006)
	40, 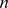 = 14	0.45 (0.17)	0.072 (0.006)	0.066 (0.019)	0.664 (0.063)	0.079 (0.007)	0.151 (0.035)	0.007 (0.004)
	60, 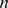 = 13	0.40 (0.13)	0.079 (0.022)	0.068 (0.018)	0.547 (0.090)	0.100 (0.016)	0.077 (0.033)	0.002 (0.002)
	80, 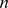 = 8	0.28 (0.08)	0.102 (0.040)	0.094 (0.027)	0.479 (0.161)	0.132 (0.024)	0.048 (0.006)	0.000 (0.000)
	100, 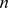 = 9	0.11 (0.06)	0.108 (0.027)	0.119 (0.033)	0.447 (0.126)	0.150 (0.031)	0.049 (0.011)	0.000 (0.000)
P2	20, 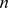 = 4	0.36 (0.10)	0.072 (0.011)	0.169 (0.035)	0.546 (0.052)	0.115 (0.022)	0.255 (0.060)	0.007 (0.009)
	40, 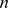 = 5	0.48 (0.25)	0.069 (0.013)	0.162 (0.053)	0.523 (0.141)	0.101 (0.027)	0.210 (0.074)	0.004 (0.005)
	60, 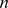 = 4	0.58 (0.03)	0.095 (0.014)	0.223 (0.038)	0.592 (0.131)	0.095 (0.009)	0.124 (0.034)	0.001 (0.002)
	80, 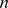 = 5	0.77 (0.30)	0.133 (0.021)	0.277 (0.050)	0.644 (0.061)	0.108 (0.020)	0.071 (0.010)	0.001 (0.001)
	100, 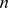 = 5	0.38 (0.12)	0.138 (0.015)	0.357 (0.119)	0.430 (0.154)	0.168 (0.040)	0.067 (0.009)	0.000 (0.000)
P3	20, 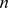 = 2	1.82 (0.04)	0.048 (0.014)	0.044 (0.006)	0.404 (0.023)	0.102 (0.029)	0.147 (0.061)	0.010 (0.006)
	40, 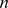 = 2	1.32 (0.66)	0.059 (0.022)	0.056 (0.009)	0.408 (0.019)	0.093 (0.011)	0.066 (0.032)	0.001 (0.002)
	60, 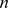 = 5	0.70 (0.30)	0.067 (0.013)	0.082 (0.010)	0.338 (0.036)	0.092 (0.012)	0.043 (0.003)	0.000 (0.000)
	80, 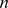 = 4	0.29 (0.06)	0.064 (0.007)	0.077 (0.015)	0.324 (0.017)	0.087 (0.009)	0.050 (0.008)	0.000 (0.000)
	100, 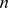 = 2	0.14 (0.11)	0.088 (0.034)	0.077 (0.046)	0.433 (0.134)	0.108 (0.037)	0.047 (0.003)	0.000 (0.000)

Shown are mean values with standard deviation in brackets.

Pigment data show that at all three process stations, phytoplankton composition was dominated by haptophytes (Table 4), predominantly *Phaeocystis* spp. At P1, dinoflagellates (25.6%) were the next in abundance while other phytoplankton groups representing between 1 and 9% of the community composition. Microscopy analysis revealed that dinoflagellates at P1 consisted mostly of individuals smaller than 10 *µ*m [40]. Community structure at P2 was similar to P1 except that the percentage of diatoms (22.5%) were two-fold more than dinoflagellates (11.0%). At this station, centric and pennate diatoms were observed to be equally distributed in which *Fragilariopsis* spp., *Pseudo-nitzschia* spp., *Chaetoceros* spp., and *Thalassiosira* spp. were the common species [40]. Other groups were represented by an abundance of 1–3%. Diatoms were observed in the deep chlorophyll maximum (DCM) just below the 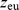. The greater abundance of diatoms probably corresponded to the occurrence of higher silicate concentration (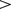 5 *µ*mol L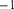) at this site. The third process station P3 was characterized by a more even distribution of haptophytes (33.2%), euglenophytes (21.3%), dinoflagellates (18.2%), and prasinophytes (11.6%). P3 also recorded the highest percentage of cyanobacteria (8.6%) compared to P1 (2.1%) and P2 (1.0%) (Table 4). Among the three stations, P3 was the most diverse in terms of species richness with the highest number of phytoplankton taxa (129 identified taxa), followed by P1 (92 identified taxa), and P2 (84 identified taxa) [40].

**Table 4:** Contribution (%) of each phytoplankton group to the total chlorophyll integrated to 120 m at each station.

Station	Prasino-phytes	Chloro-phytes	Eugleno-phytes	Crypto-phytes	Diat-oms	Dino-flag-ellates	Hapto-phytes	Cyano-b-acteria
P1	8.1	4.1	8.6	1.2	5.2	25.6	45.1	2.1
P2	1.2	0.2	2.7	1.8	22.5	11.0	59.6	1.0
P3	11.6	2.3	21.3	2.1	2.7	18.2	33.2	8.6

## Discussion

### Photoacclimation of Phytoplankton in the SAZ and PFZ

Photoacclimation in phytoplankton consists of a suite of coordinated adjustments in photophysiology and biochemistry that involves the balancing of light absorption with carbon fixation. In this study, pigments, photosynthetic and fluorescence parameters exhibited a close relationship with underwater light intensity, in which phytoplankton were exposed to a dynamic light regime from surface *in situ* PAR (200 to 700 *µ*mol photons m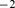 s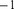), to 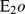 (23.1 to 36.2 mol photons m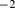 d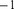), and 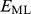 (7.6 to 12.4 mol photons m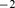 d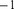). High values of 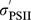 (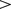 600 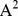 quanta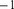) observed when the effect of *in situ* irradiance could be neglected, suggest that phytoplankton on average spent more time in a relatively low irradiance environment, which corresponds to low 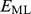 levels observed in this study. The acclimation mechanism is likely to have increased photosynthetic pigment per cell corresponding to an increase in 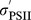 (Figure 2E) and associated decrease in 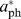 (Figure 7) below the mixed layer compared to the surface. This occurs when cells are acclimated to low irradiances, leading to subsequent increase in pigmentation and less efficient absorption per mass of pigment [41].

Low 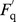/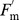 and 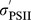 values observed in response to high *in situ* irradiance at the surface at all stations can be linked to the effects of photochemical quenching (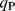) and non-photochemical quenching (NPQ) [32], [42]. The 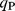 is associated with charge separation in the PSII reaction centre, whereas NPQ is a photoprotective response that is activated when irradiance exceeds the photosynthetic capacity of the cell. NPQ reduces fluorescence and encompasses a variety of processes that dissipate part of the excessive excitation energy and reduce the excitation pressure on PSII [43], [44], [45]. One process of NPQ is a rapid response (seconds to mintutes) to saturating irradiance and is dependent on thylakoid proton gradient and the operation of xanthophyll pigments cycling. Other NPQ processes are slower in response (minutes to hours) and could be due to state transition or repair of inactive PSII reaction centres [43], [46]. Rapid NPQ is an essential response to fluctuations in irradiance as it provides a rapid switch between light harvesting and thermal energy dissipation via rapid conversion of xanthophyll cycle pigments. This reduces the risk of photoinhibition and ensures optimal photosynthesis under dynamic irradiance condition [14], [43].

The major photoprotective xanthophyll cycle pigments in haptophytes, diatoms, and dinoflagellates, which were the main communities found in this study, are epoxidated diadinoxanthin (dd) and de-epoxidated diatoxanthin (dt) [14], [47]. Under low irradiance, dd assists in light harvesting by transferring energy to chlorophylls and rapid conversion from dd to dt occurs mainly when cells are exposed to excessive irradiance. This can be seen in the vertical pattern of dd+dt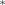 and dt/(dd+dt) (Figure 6A & B) in which high ratios of dt/(dd+dt) corresponded to high *in situ* PAR at P1, P3, and stations between P2 and P3. This shows that the low light acclimated cells were able to rapidly deploying NPQ via xanthophyll cycling to shield against excessive irradiance. A similar trend displayed in 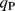 (Figure 6C), 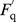/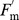 and 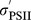, further indicates that rapid onset of NPQ was taking place in response to excessive irradiance. Pigment gradients with depth were present at all stations and the fluorescence parameters demonstrate that phytoplankton were optimizing their pigment composition to acclimate to changes in the light climate. This is consistent with studies on diatoms and haptophytes exposed to different light regimes, in which cells treated with dynamic irradiance were found to acclimate to lower irradiance than cells grow under constant irradiance [17], [18].

### Influence of Iron and Temperature on Photoacclimation

High 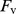/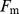, chl 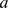 concentrations, production rates [26], [30] and low concentrations of dd+dt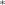 observed at P3 show that environmental conditions were favorable to phytoplankton at this station, which could be due to high iron concentration (0.45 nmol L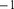) [25], [24]. At P1 and stations closer to Tasmania, photoacclimation was expressed by reduced pigment concentrations, increased 1/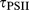 and higher 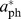 at the surface. At these stations, the photoacclimation mechanisms were effective in shifting the value of 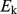 to partially track changes in ambient irradiance. High values of 1/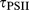, which demonstrate that there was an excess PSII capacity, could have maintained maximum carbon fixation rates even if some of the PSII reaction centers (RCIIs) were photoinhibited when they were mixed to the surface [35]. This may explain the relatively high production at these stations that were comparable to the production rates at P3, although the chl 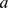 concentrations were lower than at P3 [26].

Alternatively, a large fraction of reduced RCIIs may have increased NPQ, which could have reduced the risk of photodamage [48] as shown by high surface dt/(dd+dt) at this station. Furthermore, when a large proportion of RCIIs are reduced, energy dissipation by quenchers within the antenna may be enhanced by the increased probability of the excitons populating the antenna, as shown in the depression of 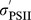, when most of the RCIIs are reduced (low 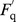/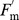 and 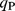). Thus cells were apparently capable of dissipating excessive excitation energy when exposed to high irradiance at the surface and were able to recover from excessive irradiance when the effect of irradiance was negligible, as indicated by high 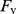/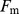 below the mixed layer.

In the case of P2, dd+dt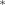 ratios were high, especially within the mixed layer, when both 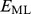 (7.6 mol m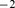 d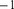) and *in situ* irradiance (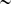
200 *µ*mol m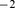 s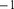) were lowest compared to P1 and P3. This suggests that xanthophyll pigments played a major role in the photoacclimation of phytoplankton in this region. Laboratory investigations of haptophytes and diatoms grown under iron limited condition have shown that cells produced more dd as a photoprotective measure when suffered from iron deficiency [13], [14], [20]. A significant negative correlation between dd+dt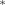 and dFe observed in this study supports that this is the case, with P2 recording the lowest dFe concentrations (0.21 nmol L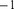). Alternatively, high dd+dt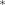 could be due to a reduction in chl 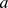 concentration as a result of low iron concentration. This would also suggests that the role of xanthophyll pigments is important in a dynamic light climate when there is a deficiency in photosynthetic pigment such as chl 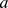 to process incoming irradiance. Having higher photoprotective/photosynthetic pigment ratio is a more cost-effective method especially for low light acclimated cells living in a nutrient depleted environment as xanthophyll pigments could protect cells from photodamage under super saturating irradiance as well as assisting in light harvesting during low light.

Photosynthetic carbon fixation rates of phytoplankton in the low nutrients open ocean have been shown to saturate at relatively low irradiances (100–300 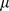
mol photons m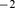 s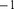) compared with the maximum surface irradiances (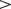 1000 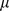
mol photons m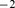 s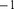) [49], [50]. Low light acclimated phytoplankton residing in low iron environments such as at P2, especially when exposed to excessive irradiance at the surface, are therefore challenged by maintaining high photosynthetic efficiency while preventing photodamage. Below the mixed layer and 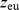, when the effect of NPQ could be neglected, high 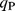 values indicate that a large fraction of RCIIs were oxidized but that the proportion of functional RCIIs remained low as indicated in low values of 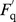/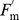. This suggests that a large proportion of RClls were not functioning. Low night values of 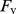/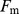 (Figure 3B) within and below the mixed layer further demonstrate that this could be the case. Photoautotrophs found in low iron regions have shown to develop a mechanism to cope with iron limitation by reducing the amount of PSI relative to PSII, as observed in cyanobacteria [11], and diatoms [12]. Low levels of electron acceptors downstream of PSII (e.g. cyt 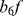 and PSI) would restrict the flow of electrons away from PSII during light exposure which could lead a big proportion of functional RCIIs remaining in a reduced state, and therefore resulting in photoinhibition or photodamage. Furthermore, relatively high values of dt/(dd+dt) observed below the mixed layer at P2 could be a result of chlororespiration, activated when phytoplankton were exposed to prolonged darkness [51].

In addition, low water temperature could have further worsened the impact of iron limitation on cells at P2. A significant negative correlation (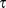 = −0.41, 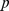 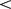 0.05, 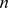 = 40) observed between dd+dt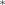 and temperature indicates that cells were probably producing more photoprotective pigments under suboptimal temperature. As a significant positive relationship (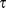 = 0.48, 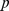 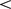 0.05, 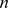 = 84) was observed between dFe and temperature, direct comparison on the influence of temperature on dd+dt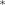 and 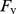/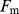 is restricted to P1 and P2, in which both stations reported similar dFe concentrations: 0.27 nmol L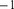 at P1 and 0.21 nmol L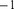 at P2. In this study, the temperature at P2 were around 4 
C, whereas the temperatures at P1 were around 12 
C. Temperature has been shown to play an important role in controlling nutrient uptake and growth of phytoplankton in the Southern Ocean [52], [53], [54]. Reduced photosynthesis, growth rate, and enzyme activity are amongst the negative impacts experienced by phytoplankton inhabiting low temperature waters [55], [56]. [53] observed a positive relationship between growth rate, nutrient uptake rate and temperature when cells at ambient temperatures ranging from 2 to 6 
C were exposed to an increase in temperature of 3 to 6 
C higher than ambient. It has been suggested that the iron requirement in the electron transport system and in nitrate- and nitrite-reductases could be elevated as a consequence of increasing the number of enzyme active-sites [53].

Recent study on the interactive effects of iron and temperature on phytoplankton in the Ross Sea demonstrated that a combined increased in iron and temperature greatly enhanced cellular abundance, phyisiology and nutrient drawdown, whereas individual increment in either parameter had only a minor effect [54]. Phytoplankton grown at low temperature in Antarctic waters were also found to have continuously limited linear electron transport due to low RUBISCO activitiy efficiency and slower metabolic repair activity [14]. This would increase the risk of photodamage for low light acclimated phytoplankton, if metabolic repair activity is not able to cope with excessive irradiance, especially in summer. Recent experiments have showed that cells with higher concentration of xanthophyll cycle pigments possessed stronger capacity to develop fast NPQ to avoid photodamage [14]. Despite exposed to lowest 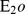 (23.1
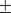
14.1 mol photons m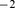 d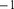) and 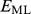 (7.6
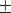
5.7 mol photons m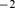 d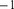), higher concentration of dd+dt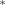 at P2 than P1 suggests that under iron limited, low temperature conditions phytoplankton communities at P2 probably relying on high abundance of xanthophyll cycle pigments to shield itself from excessive irradiance.

Additionally, low temperature has been considered to cause a time lag in the response of 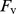/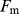 to iron addition in the Southern Ocean [15]. [15] observed that the response of 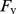/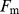 after iron enrichment was five-fold slower than iron enrichment experiments carried out in the subarctic and equatorial Pacific. In this study, 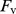/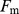 correlated positively with temperature (Table 2) which indicates that the photochemical efficiency of PSII was more efficient at higher temperature. Lab experiments have showed that PSII repair cycle is more efficient at higher temperature [14]. Hence, low values of night-time 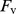/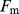 and from below the mixed layer at P2 could be caused by a combination of low dFe concentrations and suboptimal temperature. Although the mean mixed layer dFe concentrations at P1 were 0.27 nmol L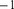, which was slightly higher than at P2 (0.21 nmol L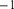), higher temperature could have maintained excess PSII capacity as indicate in high values of 1/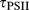 and higher electron transport rate at P1.

It is known that photophysiological response of phytoplankton varies amongst phytoplankton, in particular under the influence of iron limitation. The results of several studies on Southern Ocean diatoms and haptophytes demonstrate that under dynamic irradiance condition, lower growth rate and an enhancement in photoprotective mechanisms were observed in iron-limited diatoms. In contrast, haptophytes were able to efficiently utilize electrons flowing through PSII for carbon fixation and could maintain higher growth rate [20], [57]. Thus, in addition to iron limitation, higher concentration of photoprotective pigments and low growth rates at P2 could be due high contribution of diatoms at this site. Relatively little is known about the influence of iron limitation on dinoflagellates in the Southern Ocean and merit future investigation. Furthermore, 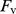/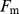 values also contain taxonomic signatures [58], [59], [60]. 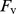/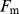 values have been observed to reach 0.65–0.7 in some species of diatoms [59] and chlorophytes [61] grown under nutrient-replete condition, whereas lower 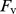/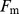 values (0.3–0.4) were recorded for some species of pelaglophytes and prasinophytes [59]. However, in iron-limited high nitrate low chlorophyll regions such as the Southern Ocean, physiological stress often outweighed taxonomic influence on 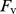/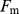 [59] as has seen reported in several iron fertilization experiments in which an increase in 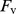/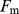 was observed upon iron relief [27]. Despite higher abundance of diatoms at P2 which should exhibit higher 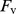/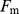, low 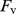/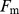 further suggested that phytoplankton at P2 suffered from iron deficiency.

It should also be noted that there is a time difference of about three weeks in between the sampling of P1 and P3 which could hamper direct comparison of data. Satellite images showed that P1 was sampled at the approximate time of the peak chlorophyll bloom, while P3 was sampled on a still rising chlorophyll bloom. Chlorophyll in the vicinity of P3 increased by 20% two weeks after the region was sampled [62]. However, cell senescence was observed at P3, while phytoplankton populations at P1 were relatively healthy [40]. This could explain similar production rates observed in both stations, despite P3 having higher chlorophyll concentration [26].

Our results demonstrate that beside iron and light, temperature could also play role in controlling phytoplankton photochemistry in the SAZ and PFZ. Suboptimal temperature could have worsened the situation for iron-stressed phytoplankton communities at P2 in the PFZ, causing cells to increase the ratio of photoprotective/photosynthetic pigments to maintain their metabolic processes under dynamic irradiance condition, and resulted in low biomass and productivity. In contrast, phytoplankton communities at P1 in the warmer SAZ-west were able to maintain efficient carbon fixation under higher irradiance condition, despite having similar dFe and chl 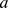 concentrations as P2. Study by [63] from the same cruise observed an increased in 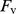/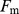 and chl 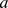 after phytoplankton community were enriched with additional iron concentrations at P3. Although P3 recorded the highest iron concentration amongst the three regions, it is believe that phytoplankton community in all three process stations were iron limited. Therefore, if the extension of warmer waters carried by the EAC persists further south, increase in water temperature together with higher iron input from the subtropical waters to the SAZ-west and probably the PFZ are likely to enhance photosynthesis in these regions. However, other factors such as thermal stratification could prevent the mixing between surface and nutrient-rich bottom waters, remains uncertain. Hence, future investigations should focus on complex interactions between phytoplankton (both physiologically and community) and a variety of physical, chemical properties, rather than focusing on a few variables.

## Supporting Information

Table S1 **Summary of sampling locations and date for FRR fluorometer deployment during the SAZ-Sense study.**
(PDF)
