# Supplementary material for: Response of Phytoplankton Photophysiology to Varying Environmental Conditions in the Sub-Antarctic and Polar Frontal Zone
Source: PLoS One. 2013 Aug 19;8(8):e72165. doi: 10.1371/journal.pone.0072165 (PMC3747055; doi:10.1371/journal.pone.0072165)
Supplement: Table S1 — Summary of sampling locations and date for FRR fluorometer deployment during the SAZ-Sense study. (PDF) [file pone.0072165.s001.pdf]

Table S1. Summary of sampling location and date for FRR fluorometer deployment during the SAZ-Sense study.

Station*	CTD	Date, 2007 (UTC)	Time (UTC)	Latitude (°S)	Longitude (°E)
1	2	19-Jan.	14:15	43.85	144.70
2	4	20-Jan.	01:50	45.00	142.98
3	6	21-Jan.	00:50	46.00	141.30
4 (P1)	10	22-Jan.	01:50	46.32	140.63
4 (P1)	12	22-Jan.	16:40	46.39	140.48
4 (P1)	18	24-Jan.	02:41	46.50	140.31
4 (P1)	20	24-Jan.	22:25	46.55	140.65
4 (P1)	21	25-Jan.	00:43	46.55	140.63
4 (P1)	22	25-Jan.	03:26	46.55	140.63
4 (P1)	23	25-Jan.	06:18	46.55	140.66
4 (P1)	24	25-Jan.	09:35	46.56	140.64
4 (P1)	25	25-Jan.	12:32	46.57	140.63
4 (P1)	26	25-Jan.	15:30	46.57	140.66
4 (P1)	27	25-Jan.	18:35	46.58	140.63
4 (P1)	28	25-Jan.	21:30	46.64	140.62
5	35	29-Jan.	11:15	49.00	143.00
6 (P2)	39	01-Feb.	05:57	54.00	145.87
6 (P2)	54	04-Feb.	03:51	54.18	146.50
6 (P2)	56	04-Feb.	09:35	54.18	146.51
6 (P2)	57	04-Feb.	17:47	54.26	146.76
7	60	06-Feb.	15:10	53.01	146.83
8	61	07-Feb.	03:00	52.01	147.71
9	63	07-Feb.	14:01	50.93	148.59
10	65	08-Feb.	03:30	50.00	149.44
11	68	08-Feb.	21:30	49.00	150.38
12	69	09-Feb.	04:58	48.01	151.22
12	70	09-Feb.	11:21	48.03	151.22
13	72	09-Feb.	23:52	47.00	152.08
14	73	10-Feb.	06:10	45.99	152.91
17 (P3)	77	11-Feb.	01:46	45.55	153.18
17 (P3)	82	11-Feb.	20:16	45.52	153.24
17 (P3)	83	12-Feb.	02:40	45.44	153.29
17 (P3)	86	12-Feb.	20:25	45.46	153.35
18	87	13-Feb.	02:27	44.75	153.01
19	88	13-Feb.	06:15	45.11	153.24
20	91	13-Feb.	22:10	44.94	152.41
21	94	14-Feb.	07:49	45.23	152.76
22	95	14-Feb.	10:55	45.27	152.97

* Process stations are showed in brackets.
